# Plasma cortisol and faecal cortisol metabolites concentrations in stereotypic and non-stereotypic horses: do stereotypic horses cope better with poor environmental conditions?

**DOI:** 10.1186/1746-6148-9-3

**Published:** 2013-01-07

**Authors:** Carole Fureix, Haïfa Benhajali, Séverine Henry, Anaelle Bruchet, Armelle Prunier, Mohammed Ezzaouia, Caroline Coste, Martine Hausberger, Rupert Palme, Patrick Jego

**Affiliations:** 1 Université Rennes 1 UMR CNRS 6552 Ethologie Animale et Humaine, Campus de Beaulieu bâtiment 25, 263 avenue Général Leclerc, Rennes Cedex, 35042, France; 2 INRA, UMR1348 Physiologie, Environnement et Génétique pour l'Animal et les Systèmes d'Elevage, Saint-Gilles, 35590, France; 3 Haras national de Sidi Thabet, Thabet, 2020, Tunisia; 4 University of Veterinary Medicine, Department Natural Sciences Biochemistry, Veterinär-Platz 1, Vienna, A-1210, Austria; 5 Current address: University of Guelph, Animal and Poultry Science department, Animal and Animal Behaviour and Welfare group, 50 Stone Road East, Building #70, Guelph, Ontario, N1G 2W1, Canada

**Keywords:** Stereotypic behaviours, Cortisol, Faeces, Plasma, Coping hypothesis, Horse

## Abstract

**Background:**

Stereotypic behaviours, *i.e.* repetitive behaviours induced by frustration, repeated attempts to cope and/or brain dysfunction, are intriguing as they occur in a variety of domestic and captive species without any clear adaptive function. Among the different hypotheses, the coping hypothesis predicts that stereotypic behaviours provide a way for animals in unfavourable environmental conditions to adjust. As such, they are expected to have a lower physiological stress level (glucocorticoids) than non-stereotypic animals. Attempts to link stereotypic behaviours with glucocorticoids however have yielded contradictory results. Here we investigated correlates of oral and motor stereotypic behaviours and glucocorticoid levels in two large samples of domestic horses (*N*_Study1_ = 55, *N*_Study2_ = 58), kept in sub-optimal conditions (*e.g.* confinement, social isolation), and already known to experience poor welfare states. Each horse was observed in its box using focal sampling (study 1) and instantaneous scan sampling (study 2). Plasma samples (collected in study 1) but also non-invasive faecal samples (collected in both studies) were retrieved in order to assess cortisol levels.

**Results:**

Results showed that 1) plasma cortisol and faecal cortisol metabolites concentrations did not differ between horses displaying stereotypic behaviours and non-stereotypic horses and 2) both oral and motor stereotypic behaviour levels did not predict plasma cortisol or faecal cortisol metabolites concentrations.

**Conclusions:**

Cortisol measures, collected in two large samples of horses using both plasma sampling as well as faecal sampling (the latter method minimizing bias due to a non-invasive sampling procedure), therefore do not indicate that stereotypic horses cope better, at least in terms of adrenocortical activity.

## Background

Stereotypic behaviours are repetitive behaviours induced by frustration, repeated attempts to cope and/or brain dysfunction [1,2]. Stereotypic behaviours typically appear in sub-optimal life conditions, *i.e.* known or believed to be aversive, *e.g.* physical confinement, social isolation and/or food deprivation. Why and how such behaviours arise nevertheless remains a highly debated issue. One explanation, commonly known as the “coping hypothesis”, is that stereotypic behaviours may help the animal to “cope” with unfavourable conditions, by providing an “enrichment” in the sub-optimal domestic situations [2] or by counteracting physical discomfort [3]. In striped mice *Rhabdomys*, stereotypic animals even have a better reproductive output [4], suggesting that some stereotypic behaviours may have beneficial effects. In horses, stereotypic mares however exhibit lower reproductive success [5], and at present, the coping function of stereotypic behaviours remains a highly debated issue. According to the coping hypothesis, individuals that display stereotypic behaviours are expected to have lower physiological stress levels (commonly assessed by measuring glucocorticoids concentrations) than non-stereotypic animals in the same sub-optimal environment. Attempts to link stereotypic behaviours with glucocorticoids however have yielded contradictory results. For example in horses, McBride and Cuddeford [6] report higher plasma cortisol (pC) levels immediately prior to a crib-biting bout, followed by a significant reduction post-crib-biting, suggesting that this stereotypic behaviour may have a coping function to reduce stress levels. On the other hand, Pell and McGreevy [7], Clegg et al. [8] and more recently Hemmann et al. [9] report on the same species no significant differences in plasma and salivary cortisol levels between stereotypic and non-stereotypic horses (see [10] for similar results in pigs and [11] in margays *Leopardus wiedii*). In contrast, McGreevy and Nicol [12] and Bachmann et al. [13] report even higher basal plasma cortisol concentrations in adult stereotypic horses than in control non-stereotypic horses (see [14] for similar results in mink).

Here we investigated specific correlates of oral and motor stereotypic behaviours and glucocorticoid levels in two large and very different samples of domestic horses kept in sub-optimal conditions and already known to experience poor welfare states [15-19]. We discuss two studies, both involving equine facilities where horses were kept in social isolation (*i.e.* boxes) and experienced time-restricted feeding practices, two factors known to trigger stereotypic behaviours *e.g.*[18,20,21]. These two studies were complementary. Study 1 (N = 55, 41 geldings, 14 mares, 5-20-year old) was performed on a working riding school population (of mostly French Saddlebred), already known to experience work-related disorders (*i.e.* vertebral problems [17,22]). Study 2 involved 58 purebred Arab brood mares (4-20-year old) all housed in the same facility where the routine did not enable the horses to be turned out in paddock, and where mares had already been shown to experience poor welfare, *e.g.* altered time budgets [15] and impaired reproductive success [5]. Each horse was observed in its box using focal sampling (study 1, 30 minutes in total per horse) and instantaneous scan sampling (study 2, 92 scans per horse). The oral and motor repetitive behaviours observed (Table 1), long termed “stereotypy” or “stereotypic behaviours” either in the litterature *e.g.*[6,7,9,23], have all been previously described in horses (review in [24]). According to a recent re-definition of terms [1,2], “stereotypy” is now reserved for a sub-class of highly predictable forms of repetitive behaviours caused by particular types of brain dysfunction [25], a criterion not demonstrably met/ investigated to date for all the observed behaviours. In addition to the “classical” repetitive behaviours, following previous studies performed in other species and in horses, repetitive licking/biting (walls, grids, feeding trough) were recorded as further abnormal repetitive behaviours (*e.g.*[3,26]). Note that we use here the term “stereotypic behaviours” as a broad descriptive term encompassing all repetitive behaviours observed, as they all typically appear in captive sub-optimal conditions that induce motivational frustration and/or physical discomfort. Plasma sample collection involves handling of the animals and can be stressful, which may influence the results *e.g.*[27,28]. Thus we used plasma (collected in study 1; two times per horse between 18:00 and 19:00 over 2 consecutive days; data obtained in the morning being less reliable, see methods) and also faecal samples (in both studies), the latter being a totally non-invasive method already well validated and used in horses *e.g.*[29-33], in order to assess cortisol levels. Furthermore, faecal cortisol metabolites (fCM) concentrations reflect an average level of circulating cortisol over a long period rather than a point in time sample. Therefore it provides a more accurate assessment of long-term cortisol levels than blood samples, which are highly dependent on the pulsatile secretion of glucocorticoids [33-36]. Faecal samples were collected between 12:00 and 13:00 three times per horse on three different days in study 1, and once between 08:00 and 10:00 in study 2. The coping hypothesis generates the following predictions. In these sub-optimal life conditions, horses displaying stereotypic behaviours would have lower pC and fCM concentrations than non-stereotypic horses. Moreover, within the sample of stereotypic horses, those with higher levels of stereotypic behaviours would have lower pC and fCM concentrations. Oral and motor stereotypic behaviours can have different, though non-mutually exclusive, aetiologies, which can be *e.g.* gastric inflammation for oral stereotypic behaviours [37], motivational frustration for social interaction and/or confinement for motor stereotypic behaviours [20]. Therefore oral and motor stereotypic behaviour levels were considered separately.

**Table 1:** Type (oral/motor), name and description of stereotypic behaviours observed

**Type**	**Name**	**Description**
Oral	Cribbing	The horse grasps a fixed object with its incisors, pulls back and draws air into its oesophagus while emitting a characteristic pharyngeal grunt.
	Lip play	The horse moves its upper lip up and down without making contact with an object, or the horse smacks its lips together.
	Tongue play	The horse sticks out its tongue and twists it in the air.
	Lip or teeth rubbing	The horse rubs its upper lip or its upper teeth repetitively against the box wall.
	Repetitive licking/biting	The horse licks or bites the box walls, box grids or external part of the feeding trough.
Motor	Head shaking and nodding	The horse bobs its head repeatedly up and down or tosses its head in recurrent and sudden bouts.
	Weaving	The horse sways laterally, moving its head, neck, forequarters and sometimes hindquarters.
	Box walking	The horse paces a fixed route around the stall.
	Door kicking	The horse kicks the door of the box repeatedly with its forelegs.

Adapted from [3]**,**[5]**,**[24]**,**[26]**].**

## Results

### Study 1

Stereotypic behaviours were observed in 65% of the horses in a total of 30 minutes of observation (9/12 horses in school 1, 18/26 in school 2 and 9/17 in school 3, chi-square test: *X 2 2* = 1.82, *P* = 0.50) with a median frequency of 0.03 times per min (Q1 = 0.0, Q3 = 0.20, range: 0–0.8). Stereotypic behaviours were distributed as follows: repetitive trough licking (15 horses), head shaking and nodding (14 horses), lip play (13 horses), repetitive object biting (10 horses), repetitive wall licking (7 horses), lip or teeth rubbing (4 horses), weaving (3 horses) and cribbing (1 horse) (median frequencies and ranges shown in Table 2). Eighteen horses (33%) exhibited more than one stereotypic behaviour. PC concentration varied from 2.5 to 40.3 ng/mL (Med = 11.0, Q1 = 6.0, Q3 = 20.7). Concentrations of fCM varied from 1.6 to 13.1 ng/g (Med _sample 1_ = 4.2, Q1 = 3.5, Q3 = 6.0). No difference appeared according to age (plasma: *F 1, 48* = 0.75, *P* = 0.39; fCM: *F 1, 46* = 3.55, *P* = 0.07). No difference appeared according to sex for fCM concentrations (*F 1, 46* = 0.37, *P* = 0.54); females however had higher pC concentrations than geldings (Med _Females (*N* = 14)_ = 17.3, Q1 = 12.8, Q3 = 24.0, Med _Geldings (*N* = 41)_ = 6.8, Q1 = 6.0, Q3 = 14.6, *F 1, 48* = 8.29, *P* = 0.006). Interestingly, plasma cortisol concentration predicted fCM concentrations: the higher the plasma cortisol concentration was, the higher the fCM concentration was (*F 1, 53* = 36.43, *P* = 0.0001).

**Table 2:** Median frequency per minute and range (minimum – maximum) per type of each stereotypic behaviour observed in horses from riding school (study 1)

**Name of the stereotypic behaviour**		**Median frequency per minute, range (minimum – maximum)**
Repetitive licking/ biting	Feeding trough (*N* = 15 horses)	0.03 (0.03 – 0.30)
	Object biting (*N* = 10)	0.08 (0.03 – 0.17)
	Wall (*N* = 7)	0.10 (0.03 – 0.13)
Head shaking and nodding (*N* = 14)		0.10 (0.03 – 0.57)
Lip / tongue play (*N* = 13)		0.10 (0.03 – 0.43)
Lip or teeth rubbing (*N* = 4)		0.03 (0.03 - 0.07)
Weaving (*N* = 3)		0.27 (0.10 – 0.67)
Cribbing (*N* = 1)		0.03

Number in brackets following the name of the behaviour = number of horses observed performing the behaviour at least once.

**Figure 1:**
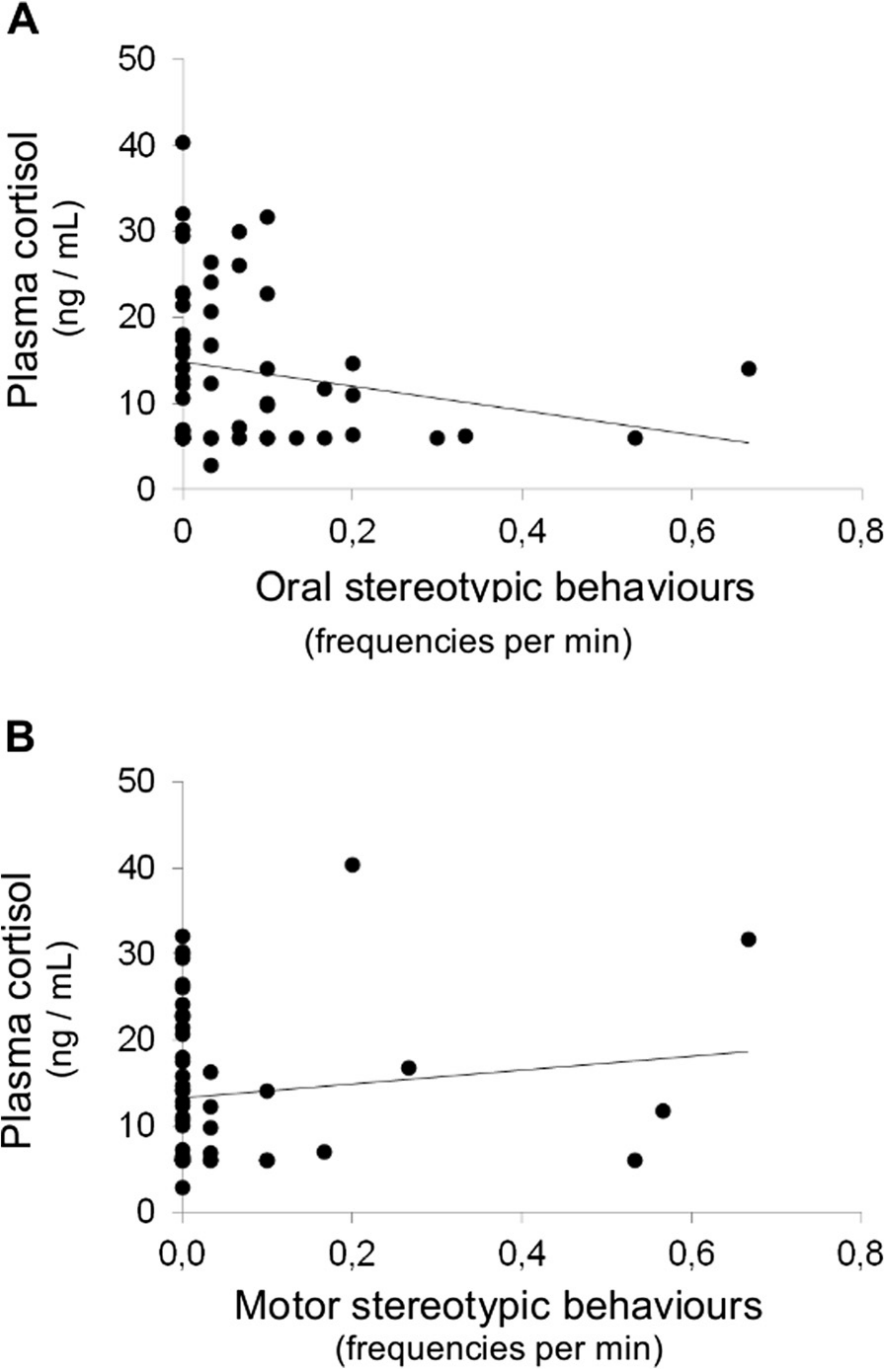
**Plasma cortisol concentrations (ng/mL) function of oral (A) and motor (B) stereotypic behaviours frequencies in horses from riding schools (study 1, N = 55).** Plasma cortisol concentrations were assessed two times per horse (between 18:00 and 19:00) and averaged. Original data are presented for clarity (plasma cortisol concentrations were Box Cox-transformed and stereotypic behaviours were log-transformed for analysis). Neither oral nor motor stereotypic behaviours frequencies predicted plasma cortisol concentrations (respectively *F 1, 48* = 1.11, *P* = 0.30 and *F 1, 48* = 0.86, *P* = 0.36).

**Figure 2:**
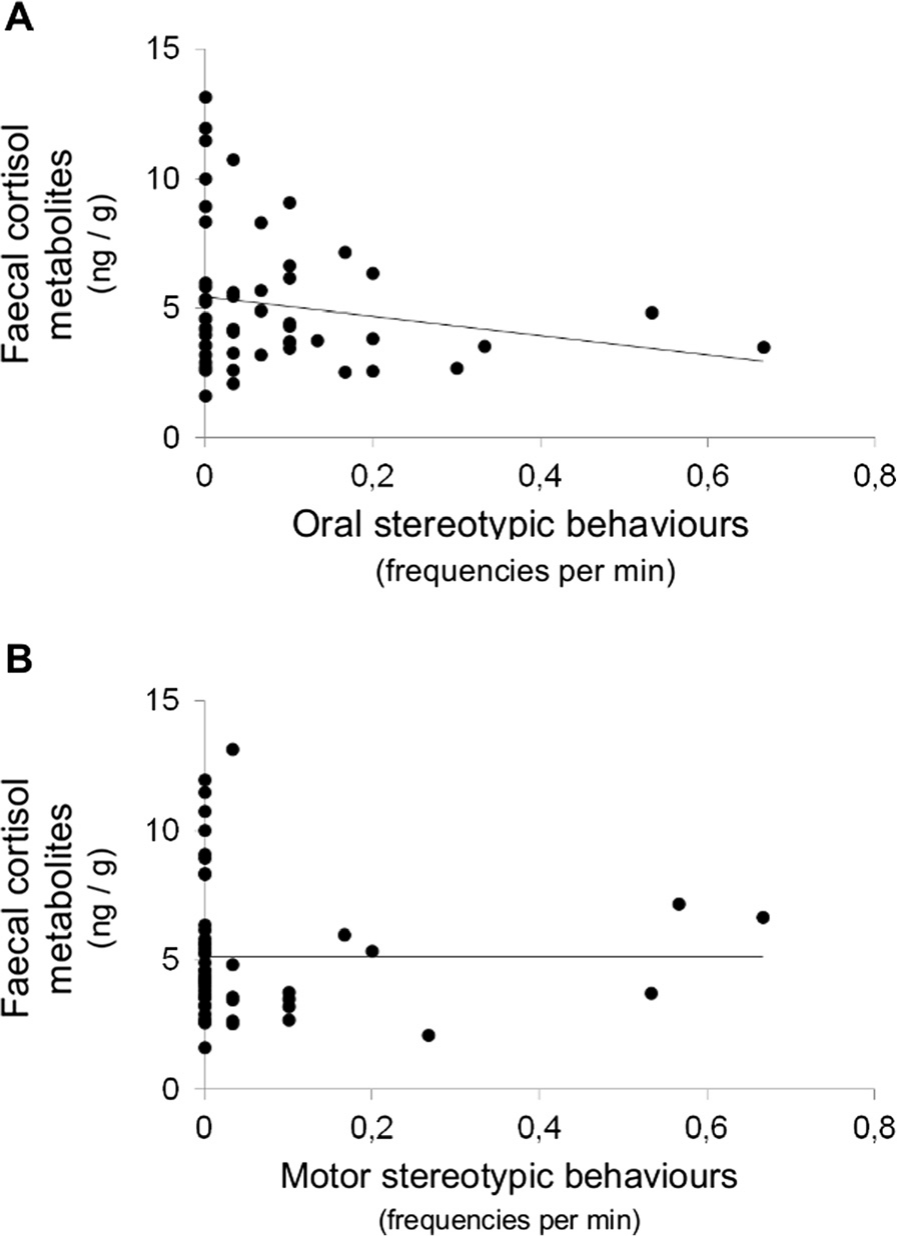
**Faecal cortisol metabolites concentrations (ng/g) function of oral (A) and motor (B) stereotypic behaviours frequencies in horses from riding schools (study 1, N = 55).** Samples were collected between 12:00 and 13:00, three times per subject: a sample on two different days, each 24 h after a day’s work and one sample 24 h after a day’s rest, then averaged. Original data are presented for clarity (data were log-transformed for analysis). Neither oral nor motor stereotypic behaviours frequencies predicted faecal cortisol metabolites concentrations (respectively *F 1, 46* = 0.52, *P* = 0.47 and *F 1, 49* = 0.23, *P* = 0.64).

Whether or not the horse displayed at least one stereotypic behaviour did not predict pC concentration (*F 1, 48* = 0.002, *P* = 0.96), and this was still true when oral (Figure 1a, *F 1, 48* = 1.11, *P* = 0.30) and motor stereotypic behaviours frequencies (Figure 1b, *F 1, 48* = 0.86, *P* = 0.36) were considered separately. Similarly, displaying at least one stereotypic behaviour did not predict fCM concentration (*F 1, 46* = 0.38, *P* = 0.54), even when oral (Figure 2a, *F 1, 46* = 0.52, *P* = 0.47) and motor stereotypic behaviours frequencies (Figure 2b, *F 1, 49* = 0.23, *P* = 0.64) were considered separately.

### Study 2

Stereotypic behaviours were observed in 24% of the horses (14/58, median frequency = 0.1, Q1 = 0.0, Q3 = 0.1 scans, range: 0–22) and were distributed as follows: weaving (9 horses), box walking (5 horses), repetitive door kicking (1 horse), head nodding (1 horse) and lip play (1 horse) (median number of scans and ranges shown in Table 3). Four horses (7%) presented more than one stereotypic behaviour. Concentrations of fCM varied from 2.4 to 37.6 ng/g (Med = 6.8, Q1 = 9.2, Q3 = 13.6). No difference appeared according to age (*F 1, 47* = 0.11, *P* = 0.74) or reproductive status (*F 1, 47* = 0.40, *P* = 0.67).

**Table 3:** Median number of scans in which a stereotypic behaviour was observed and range (minimum – maximum) per type of each stereotypic behaviour observed in brood mares (study 2)

**Name of the stereotypic behaviour**	**Median number of scans, range (minimum – maximum)**
Weaving (*N* = 9 horses)	5 (1 – 22)
Box walking (*N* = 5)	5 (2 – 8)
Repetitive door kicking (*N* = 1)	4
Head shaking and nodding (*N* = 1)	3
Lip play (*N* = 1)	1

Number in brackets following the name of the behaviour = number of horses observed performing the behaviour at least once.

**Figure 3:**
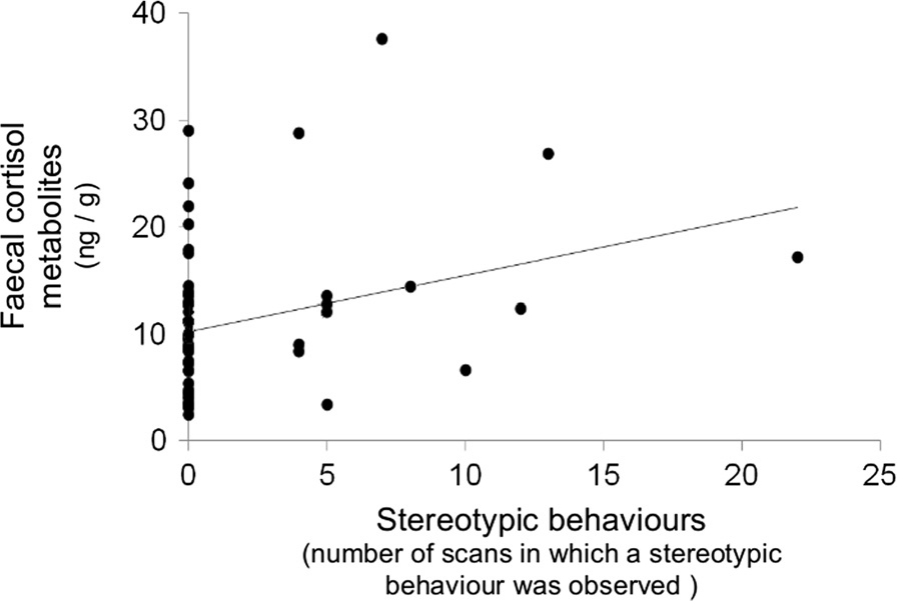
**Faecal cortisol metabolites concentrations (ng/g) function of number of scans in which a stereotypic behaviour was observed in Arab mares (study 2, N = 58).** Samples were collected between 08:00 and 10:00. Original data are presented for clarity (data were log transformed for analysis). The number of scans in which a stereotypic behaviour was observed did not predict faecal cortisol metabolites concentrations (*F 1, 47* = 0.003, *P* = 0.96).

Again, whether or not the horse displayed at least one stereotypic behaviour did not predict fCM concentration (*F 1, 47* = 0.001, *P* = 0.99), and the number of scans in which a stereotypic behaviour was observed also did not predict fCM concentrations either (Figure 3*, F 1, 47* = 0.003, *P* = 0.96).

## Discussion

Here we investigated specific correlates of oral and motor stereotypic behaviours and glucocorticoid levels in two large and different samples of domestic horses, kept in sub-optimal conditions and already known to experience poor welfare states. We used plasma and also non-invasive faecal sampling to measure cortisol levels. According to the coping hypothesis of stereotypic behaviours – *i.e.* that stereotypic behaviours are performed as a means of helping the animal to cope with sub-optimal environments by reducing stress – horses that displayed stereotypic behaviours were expected to have lower cortisol levels than non-stereotypic horses. Furthermore within the sample of stereotypic horses, those with higher levels of stereotypic behaviours were expected to have lower cortisol levels. Both pC (study 1) nor fCM (study 1 and 2) concentrations however differed between stereotypic and non-stereotypic horses, nor were they significantly predicted by stereotypic behaviour levels, even when oral and motor stereotypic behaviour levels were considered separately. Therefore, cortisol measures do not indicate that stereotypic horses cope better, at least in terms of adrenocortical activity.

Our results confirm the earlier findings of Pell and McGreevy [7], Clegg et al. [8] and Hemmann et al. [9] indicating no significant relationship between plasma/salivary cortisol levels and stereotypic behaviours in this species. Interestingly, this absence of a relationship between plasma cortisol and stereotypic behaviour levels appears to be independent of the time of plasma sampling. Indeed, Hemmann and collaborators [9] report on not significant effects of crib-biting on cortisol circadian secretion when collecting plasma every two hours for 24 h in stereotypic and control non-stereotypic horses. When added to Pell and McGreevy’s [7] and Clegg’s et al. [8] previous studies, where plasma was collected in the morning, our complementary results based on plasma samples collected in the afternoon support Hemmann and collaborators’ conclusions. Moreover, in the four previous studies [7,8,12,13] in which plasma samples were all collected in the morning, results were also contradictory, indicating that time of sampling cannot be the main explanation for results discrepancy. In this context, using faecal samples, that reflect an average level of circulating cortisol over a longer period, appears nevertheless to be a good complementary strategy.

Using faecal samples however might also raise a new methodological issue, namely a potential impact of variations in gut flora on the concentrations of cortisol metabolites. Indeed, the metabolites of cortisol that are measured in faeces are the products of extensive modification by bacteria in the gut (e.g. [35,36]). As a consequence, the composition of bacteria could influence the type and/or the quantity of hormone metabolites (discussed in [38]). Imbalance in hindgut flora (as a result of acidosis) has been reported in horses displaying crib-biting [3] and one could expect this imbalance to influence fCM concentrations in these animals compared with non-stereotypic horses and to bias the results. FCM concentrations however did not differ between stereotypic and non-stereotypic horses, nor were they significantly predicted by stereotypic behaviour levels, even when oral and also motor stereotypic behaviour levels were considered separately. To our knowledge, motor stereotypic behaviours have not been reported to be linked with gastric nor gut disorders in horses; therefore horses displaying motor stereotypic behaviours would not be expected to differ from control horses in regards to their gut flora composition. In addition, no significant relationship between stereotypic behaviours and cortisol levels appeared either when taking blood samples measuring the actual hormone (a complementary approach advised by [38]). Therefore, our results that cortisol measures do not seem to indicate that stereotypic horses cope better appear unlikely to reflect methodological bias due to sampling methods, though further research on the potential effect of gut floral on fCM concentration is warranted.

The idea that stereotypic behaviours may help animals to cope with sub-optimal environments is not new *e.g.*[39-41], but it is still a highly debated issue and evidence is sparse and contradictory. McBride and Cuddeford [6] report higher plasma concentrations in horses immediately prior to the onset of a crib-biting bout, followed by a significant reduction post- crib-biting, suggesting that stereotypic behaviours may have a coping function that reduces stress levels in the animal. According to these results, the prevention of stereotypic behaviours may then be even more stressful and should induce a rise in glucocorticoids. In order to further test this prediction, two experimental studies have examined the effects of stereotypic behaviour prevention under controlled conditions on cortisol levels in horses. However, the environmental modifications used to prevent the stereotypic behaviours (*i.e.* devices such as crib-strap and anti-weave bar, removal of both a cribbing bar and hay) induced a rise in plasma cortisol concentrations in stereotypic horses, but also in control non-stereotypic horses [6,12]. Thus, the observed rise in glucocorticoids in stereotypic horses, initially expected to reflect a stress response due to the prevention of stereotypic behaviours but also observed in non-stereotypic animals, might simply reflect the horse’s response to environmental changes as previously suggested in rodents *e.g.*[42].

Note also that, as reported in the literature, differences in cortisol levels between stereotypic and non-stereotypic horses do not support the coping hypothesis prediction. Indeed, McGreevy and Nicol [12] and Bachmann et al. [13] reported higher plasma cortisol concentrations in stereotypic horses than in non-stereotypic horses. One however may argue that higher cortisol levels in stereotypic animals could represent methodological bias, particularly when moving horses from their home stable to an experimental one and/or using frequently repeated blood sampling, which both could be stressful for the animals [27,28]. Stereotypic horses may be particularly stress-sensitive individuals and they may perceive environmental changes and blood sampling as even more stressful than non-stereotypic animals, which may have impaired the results. In this context, using faecal samples – *i.e.* a totally non-invasive measurement, yielding no bias due to sampling procedure – to assess adrenocortical activity appears to be a better strategy and reinforces the earlier findings cited above that no relationship is evidenced between stereotypic behaviours and basal glucocorticoid levels.

On the whole, our results also do not support the coping hypothesis prediction, though several explanations for these negative results can be proposed. The absence of difference between stereotypic and non-stereotypic horses might reflect equally low cortisol levels, an interpretation which would, at least partly, support a coping function of stereotypic behaviours. Bearing in mind however that horses can express poor welfare states in various ways (*e.g.* aggressiveness [17], apathy and unresponsiveness [19,43]), one may also argue that, in the experienced sub-optimal life conditions, both stereotypic and non-stereotypic horses were equally stressed, *i.e.* equally high cortisol levels.

Another possible explanation for the results is that chronic stress levels might not be accurately measured by basal cortisol concentrations *e.g.*[34,44]. Cortisol has a circadian rhythm (highest in the early morning, lowest in the evening), a phenomenon well-evidenced in horses [45-50]. Some chronic stressors have been reported to induce dysregulated pattern of hormone secretion, *e.g.* a flattened diurnal rhythm [51]. Highly frequent blood collection can be a disturbing procedure in itself and is also clearly not always practicable or possible, especially in this study performed on working horses from riding schools. Including a pC concentration assessment at least at the times where cortisol concentrations are highest and lowest (the initial protocol of study 1, but not taken due to methodological reasons, see “methods”) would allow a calculation of a slope as an indication of cortisol’s rhythm across the day, and would provide additional interesting information. Note, however, that the effect of chronic stress on glucocorticoid circadian variations is still far from straightforward, as it might vary according to the stressor and individual features [51]. Thus it would also be interesting to add other measures of coping to test further the coping hypothesis prediction.

Beyond the potential functional explanation of stereotypic behaviours proposed by the coping hypothesis, another explanation for our negative findings may be that the performance of stereotypic behaviour is not associated with stress or coping at all. Indeed, some forms of stereotypic behaviours are preservative and animals cannot stop performing them [25]; stereotypic behaviours can become habit forming (*e.g.* enhancement of habit formation in crib-biting horses [52]) and thus does not result in changes in cortisol levels; and/or the eliciting stimuli might no longer be present in the current environment *e.g.*[53]. Note however that the latter issue would be hard to test in our population since these horses are still experiencing several challenges to their welfare, such as confinement, social isolation and time-restricted feeding practices.

## Conclusions

This is, to our knowledge, the first time that a non-invasive measure of cortisol (*i.e.* without potential bias due to sampling procedure) was performed in addition to plasma cortisol analysis in order to investigate the relationship between stereotypic behaviours and adrenocortical activity in horses. The present data do not show a significant relationship between stereotypic behaviours and both pC and fCM concentrations in two large and very different groups of domestic horses kept in sub-optimal conditions and already known to experience poor welfare states. This appears to be a general trend, as neither oral nor motor stereotypic behaviours predicted glucocorticoids levels. Cortisol measures therefore do not seem to indicate that stereotypic horses cope better, at least in terms of adrenocortical activity.

## Methods

All our experiments complied with current French laws related to animal experimentation and were in accordance with the European directive 86/609/CEE. The local Ethics Committee in Animal Experiment of Rennes gave a favourable opinion to perform both studies. No licence/permit/institutional ethical approval were needed from the local Ethics Committee in Animal Experiment of Rennes (study 1) as the work respected French regulations and blood samples were obtained in presence of a veterinarian doctor. No licence/permit/institutional ethical approval were needed for study 2 according to the Tunisian regulations, as only behavioural observations and non-invasive sampling (in presence of the veterinarian doctor of the breeding facility) were performed. In both studies, animal husbandry and care were under management of the riding schools and breeding facility staff, as this experiment involved only horses “from the field” (no laboratory animals).

### Subjects

#### Study 1

Fifty-five horses (37 French Saddlebred and 18 diverse breeds and unregistered horses) from three riding schools (*N* = 12, 26 and 17 horses respectively; all horses at the three riding schools were included in the study) in the western part of France were observed between January and May 2007. Activities and housing conditions were similar in the three riding schools. In all cases, the horses were kept singly in 3 m × 3 m individual straw-bedded boxes, with solid walls between boxes (but visual contact with conspecifics was possible from the box doors). Each box was cleaned once a day (in the morning) and was equipped with an automatic drinker. Animals were fed industrial pellets (mainly composed of wheat bran, 30%; barley, 28%; flour of alfalfa, 10%; palm kernel, 10%; soya bean, 10%; oats, 6%; treacle, corn, calcium carbonate, sodium chloride, vitamins A, D and E; copper sulphate) three times a day and hay was provided *ad libitum*. Horses worked in riding lessons for 4–12 hours a week, with at least one free day each week (riding school day off, where horses from the riding school #2 were released in paddocks). Riding lessons involved children and teenagers and were related mainly to indoor (instruction) and outdoor activities, including a few competition activities. This sample included both geldings (*N* = 41) and mares (*N* = 14). They were 5 to 20 years old ($\overset{―}{X}=11.9\pm 3.5$).

#### Study 2

Fifty-eight purebred Arab mares were observed from the 30^th^ March to the 15^th^ of May 2005 at the national stallion breeding facility of Sidi Thabet, located 20 km from Tunis in Tunisia. Mares are brought to this facility every year in order to breed with the stallions housed there. None of the mares were pregnant at the time of the study, but they belonged to three different reproductive categories: foaling mares (mares mated/inseminated in the facility where we conducted this study with a foal at foot that was born and bred in the facility, *N* = 40, 5–20 years old, $\overset{―}{X}=9.8\pm 4.2$ years), non-foaling mares (with no foal at foot, *N* = 11, 5–18 years old, $\overset{―}{X}=10.2\pm 4.3$) and “maiden” mares, *i.e.* mares with no foal at foot and staying at the breeding facility for the first time (*N* = 7, 4–6 years old, $\overset{―}{X}=4.7\pm 1.0$). Reproduction management took place between 10:00 and 11:00 and included oestrus detection (by teasing every 48 hours, and rectal palpation and ultrasound), mating or inseminations and pregnancy diagnosis (ultrasound examination) [21]. Mares were housed in individual stalls where they received barley grain (4 kg/day), hay every morning and evening and some freshly cut grass once a day. The routine in this facility does not enable the horses to be turned out. Stalls (5 m × 3 m) were straw bedded and visual contact with conspecifics was possible from the stall doors (solid walls between boxes). Horses were allowed to drink about 5 min twice a day from the communal trough available outdoors. Mares were 4 to 20 years old ($\overset{―}{X}=9.28\pm 4.31$); maiden mares were younger than others (Kruskall-Wallis test: *H*_2, 58_ = 13.4, *P* = 0.01).

### Behavioural observations

These two complementary studies were part of two different research projects (one performed in 2005 and the other in 2007); behaviour sampling methods therefore differed between study 1 and study 2.

#### Study 1

Each horse was observed by a single observer (CF) in its box using a focal sampling method [54]: all occurrences of all behaviours of the focal animal were recorded continuously during 5 min sessions. Only one horse was observed at a time (*i.e.* one focal animal) and horses were pseudo-randomly assigned to observations (*i.e.* neighbours were not observed in succession). Observations were made during three periods: in the morning between 09:00 and 11:00, in the afternoon between 14:00 and 17:00 and half an hour before meal times (*i.e.* between 06:30–07:30, 11:30–12:00 or 17:30–18:00, according to school schedules). The fact that food was distributed manually (yielding more frustration, more agitation and more anticipatory behaviours than when all the horses are fed simultaneously, for instance by an automatic feeder) created a favourable situation for observing repetitive movements *e.g.*[18,20,24]. Each horse was observed during 6 sessions performed during a 10-day period (2 sessions per time period, *i.e.* 30 min in total/horse).

#### Study 2

Observations were made by a single observer (HB) every day during 46 days using instantaneous scan sampling. Twice a day (once in the morning before feeding and once in the evening after feeding), the observer walked through along the stalls and noted the behaviour of each of the mares at the instantaneous time of her passage. The time budget for each behaviour was determined as the recorded number of each behaviour divided by the total recorded number of scans in each horse. Previous observations and preliminary observations indicated that two such scans are enough to identify stereotypic animals, especially over a longer time period as was the case in this study [5].

Although we recorded all behavioural patterns in both studies, presented data are limited to stereotypic behaviours. The oral and motor stereotypic behaviours observed are reported in Table 1.

### Physiological data: adrenocortical activity

#### Plasma cortisol measurement (study 1)

We aimed to minimise the aversive effects of blood sampling, which was confirmed by the absence of any retreat behaviour of the horses. Each horse was lightly restrained by a single experimenter who was unknown to the horse (SH) and systematically given a food reward (one sugar lump) at the end of each blood sampling. Sampling was made by a single experimenter (CF) and the total duration of the procedure did not exceed one minute. Blood samples were collected from the left jugular vein two times per horse between 18:00 and 19:00 over 2 consecutive days: once after a day’s work and once after a day’s rest. The initial protocol also included morning sampling. Preliminary analysis however revealed a limitation of our method. Morning cortisol concentrations were highly influenced by the time of sampling (Fureix et al. in prep), more particularly in regards to the time passed between the dawn and the actual time of sampling (an interval which was likely to differ from January to May). We therefore excluded morning samples from analyses in the present study.

Seven ml of blood were collected in heparinised polypropylene tubes (BD Vacutainer®). Samples were kept on crushed ice until centrifugation (with a maximal delay between sampling and centrifugation of 15 min) and then aliquots of plasma were immediately placed on dry ice and stored at −20°C for further processing. Plasma cortisol levels were measured using radioimmunoassay Immunotech kits for cortisol determination (Beckmann and Coulter). These kits are usually used for measuring human plasma cortisol. We modified the manufacturer’s method so that it could be used for equine plasma that contains more interfering proteins: 1) the quantity of plasma per dose was 25 μL instead of 50 μL; 2) a two-hour preliminary incubation at 20°C between plasma and antibodies was added; 3) we used two standard curves: the first with increasing cortisol concentrations in buffer (as indicated by the manufacturer) and the second with increasing cortisol concentrations in equine plasma (diluted in a pool sample of equine plasma containing low cortisol levels). These modifications produced linear curves (log B/Bo) between 2 ng/mL and 300 ng/mL. A good linearity was observed for dilution or overload experiments. The coefficient of variation (one sample measured seven times in the same assay) was 1.37%. Note that the range of absolute pC concentrations we obtained was apparently comparable to the data reported in the literature, keeping however in mind that absolute values are highly method dependent (and thus may vary by a factor of 2, or even more).

#### Faecal cortisol metabolites measurement (study 1 and study 2)

Fresh faecal samples were collected immediately (less than 1 minute) after defecation directly from the bedding. In study 1, samples were collected between 12:00 and 13:00, three times per subject: two samples were collected on two different days, 24 h after a day’s work and, a third sample was collected 24 h after a day’s rest (taking the 24 h delay in excretion of fCM in horses into account; [34,35]). Note that plasma (see above) and faecal sampling were not time-matched in our study (*i.e.* each faecal sample was not collected 24h, which is the delay in excretion of fCM in horses [32], after each plasma sample). Therefore pC and fCM concentrations did not reflect simultaneous glucocorticoids levels, but rather provide a broader assessment of the adrenocortical activity. In study 2, only faecal cortisol metabolites were measured. A fresh faecal sample per horse was collected once between 08:00 and 10:00 immediately after defecation directly from the bedding.

Each faecal sample was then kept frozen at −20°C until further analysis. Extraction of samples followed the method described by Merl et al. [30]. Briefly, 0.5 g faeces plus 1 ml water and 4 ml methanol were vortexed for 30 minutes and centrifuged (2500 g/15 min). One ml of the supernatant was mixed with 5 ml diethylether and 0.5 ml 5% NaHCO_3_ for 10 seconds. Thereafter, 4 ml water were added, the tube was inverted four times and the aqueous phase was frozen at −24°C. Afterwards the ether phase was decanted and dried down. The extract was re-dissolved in assay buffer and the concentration of 11,17-dioxodandrostanes (11,17-DOA), a group of cortisol metabolites, was measured with an 11-oxoaetiocholanolone enzyme immunoassay (EIA), previously described [55] and successfully validated for use in horses [56].

### Data and statistical analyses

Behavioural data collected were frequencies (per min, as the total time of observation was less than one hour) of oral and motor stereotypic behaviours (study 1) and number of scans performing a stereotypic behaviour (study 2). Only one out of the 58 horses displayed an oral stereotypic behaviour (in addition to its motor stereotypic behaviours), therefore the distinction between oral and motor stereotypic behaviours was not relevant in study 2. Physiological data collected were plasma cortisol (pC, ng/mL, study 1) and/or faecal cortisol metabolites (fCM, ng/g, studies 1 and 2). Cortisol levels after a day’s work and after a day’s rest were positively correlated (Spearman correlation tests: plasma *r s* = 0.56, fCM *r s* = 0.44 to 0.69, *N* = 55, *P* = 0.001 in all cases) and no significant difference could be detected between sampling time periods (plasma: Wilcoxon test: *Z* = 0.62, *P* = 0.51, *N* = 55; fCM: Friedman test _(55, 2)_ = 4.8, *P* = 0.09). Data were therefore averaged between sampling time periods, either for plasma and fCM. Descriptive statistics are median values (Med), followed by 1^st^ (Q1) and 3^rd^ (Q3) quartiles, range.

Analyses were conducted in JMP 9.0.2. (SAS Institute Inc., Cary, NC, USA) (accepted *P* level = 0.05, two tailed tests). Horses were *a posteriori* binary classified for the analyses as “being stereotypic” (*i.e.* observed at least once performing an oral and/or a motor stereotypic behaviour shown in Table 1) or “non-stereotypic” (*i.e.* never observed performing a stereotypic behaviour). Relationships between cortisol levels (plasma cortisol or fCM concentrations) and being stereotypic or not were analysed using general linear models (GLMs), controlling for age, frequencies of oral and motor stereotypic behaviours (both type of stereotypic behaviours were considered separately as they can have different aetiologies), and other factors where appropriate, *i.e.* sex in study 1 and reproductive status in study 2. Normality and homogeneity of variance were assessed by inspection of residuals [57] and Bartlett’s test for equal variances was used where the effects of interest were categorical. Data were transformed where needed to meet the assumptions of parametric tests; all but one (namely pC concentrations, which were Box Cox-transformed) cortisol levels and stereotypic behaviour levels were log-transformed. None of the interactions were significant (*P* = 0.14 to 0.93), results will therefore not be presented here.

## Competing interests

The authors declare that they have no competing interests.

## Authors’ contributions

CF designed the study 1, collected physiological and behavioural data, performed the statistical analyses and drafted the manuscript. HB designed the study 2 and collected physiological and behavioural data. SH helped to collect the physiological data in study 1. AB, AP, ME, CC carried out plasma cortisol measurements. MH designed both studies and drafted the manuscript. RP carried out faecal cortisol measurements, helped to perform the statistical analyses, and drafted the manuscript. PJ designed the study 1. All authors read and approved the final manuscript.
